# Development and validation of a geographic search filter for PubMed for retrieving studies about Nordic countries

**DOI:** 10.5195/jmla.2026.2311

**Published:** 2026-10-01

**Authors:** Malin Barkelind

**Affiliations:** 1 malin.barkelind@ub.uu.se, Librarian, Learning and Research Support Division, Uppsala University Library, Uppsala, Sweden

**Keywords:** PubMed, information storage and retrieval, Scandinavian and Nordic Countries

## Abstract

**Objective:**

This study describes the development and validation of a geographic search filter. The filter is intended to find references on the Nordic countries in PubMed. This type of filter enables efficient systematic literature searches for evidence that is relevant for the region, and no validated filter for these countries is publicly available at the time of this publication.

**Methods:**

The Nordic countries are Denmark, Finland, Iceland, Norway and Sweden. References on one or more of these countries were identified by relative recall, using systematic and scoping reviews that did not limit searches to any geographic region but presented the countries of included studies. The references (n = 288) were equally split into a development set and a validation set. A combination of objective and subjective methods was used to identify relevant terms from the development set. The filter was then tested, first on the development set, then on the validation set. A sample of the reviews that were used to identify references for recall testing was also used to test precision.

**Results:**

The final filter surpassed the threshold recall of 90%. Recall was 94.4% for the development set, capturing 136 of 144 references, and 96.5% for the validation set, or 139 of 144. Precision was 95.8% with one false positive of 24 retrieved references.

**Conclusions:**

A validated filter has been developed that can be used to find studies on the Nordic countries when searching PubMed on topics related to human health.

## INTRODUCTION

Systematic reviews and other types of evidence synthesis rely on systematic searches to retrieve as much of the relevant literature as possible, which minimizes the review’s bias [1]. Search filters, also called hedges, are used in systematic literature searches to find literature with a common feature, such as a study design, publication type or subject area [2]. What sets search filters apart from other search strategies is that they are intended for reuse and usually systematically developed and tested.

One type of search filter is the geographic filter. To draw conclusions that are relevant for a country or region, research that is specific to that geographic location might be required. This type of filter has been developed for countries and regions such as the United Kingdom, Germany and the Organisation for Economic Co-operation and Development (OECD) countries [3–5].

In this article, I describe the development and validation of a search filter for the Nordic region. The Nordic countries include the three Scandinavian countries Denmark, Norway and Sweden, and also Iceland and Finland. The term Scandinavia is sometimes used to refer to all five countries. The Nordics also include the autonomous regions of Denmark, which are Greenland and the Faroe Islands, as well as the autonomous region Åland Islands of Finland [6]. The Nordic countries are not just a geographically cohesive area in the northern part of Europe, but also a region with similar institutions and with close connections, for example in academia, politics and economy. The countries also collaborate on issues related to public health, through the Nordic Welfare Centre [7].

Limiting the geographical scope of reviews to the Nordic countries is done in health-related research, such as epidemiology [8], qualitative studies [9] and social determinants of health [10], to mention a few recent examples. The strategies used to limit searches to the Nordic countries vary, and although the ISSG Search Filter Resource compiles a list of validated geographic filters, there are none to be found for the Nordic countries or any of the constituent countries or regions [11].

To further investigate whether other Nordic countries filters are currently available, PubMed, Scopus and Web of Science Core Collection^*^ were searched on November 13, 2025. The search strategies were inspired by the ISSG searches for existing tools in preparation of the search filter appraisal checklist [12].^†^ No search filters were found, based on title/abstract screening of all search results.

Each Nordic country’s public health authority was contacted, or when possible, information specialists working there were contacted directly, to ask if they were aware of any geographic filter for the Nordic countries. The same question was asked in online forums for librarians working with systematic searching: Bibliotekarer involvert i systematiske oversikter i Norge (BISON), Norway, and Nätverket för systematisk sökning i vetenskaplig kontext (SSIVK), Sweden. A geographic filter for the Nordic countries was identified. It was developed by Ragnhild Agate Tornes and Marita Heintz at the Norwegian Institute of Public Health [13]. It is designed for use in Medline (Ovid), Embase (Ovid), PsycInfo (Ovid), CINAHL (EBSCO), Web of Science and Scopus. Some testing of the filter has been done, but the filter is currently not validated [personal communication from M. Heinz to author, 6 Nov 2025].

The search filter that this study describes is intended for use in searches related to human health and has been developed with the purpose to be used to retrieve literature related to the Nordic countries in PubMed. PubMed is one of the most common databases to search when gathering evidence related to health, and since it is a free resource, this filter can be used irrespective of institutional subscriptions.

The aim has been to develop a balanced filter, with high recall and precision, without maximizing one or the other.

## METHODS

The method used for development and validation of the filter was adapted from the guide to develop validated geographic search filters by Ayiku et al. [14]. This method consists of five steps: (i) defining the geographic region, (ii) finding references for the region, (iii) forming the gold standard set which is split into two sets for development and validation, (iv) developing the filter using the development set and (v) validating the filter using the validation set [14].

The performance of a search filter should be tested to ensure sufficient recall and precision. Recall, also known as sensitivity, is the number of matching records out of the total number of relevant records, while precision is the number of relevant records retrieved out of the total number of matching records [2].

### Defining the Scope

The search filter developed and validated in this study is meant to retrieve references about the Nordic countries: Denmark, Finland, Iceland, Norway and Sweden.

### Gold Standard Creation

A gold standard, according to Jenkins’ definition, is the “set of relevant records identified against which the performance of the search strategy is tested and validated” [2]. In this study, a gold standard set of references was constructed, containing references that are known to be relevant to the geographic region. To create the gold standard, relative recall was used to identify the relevant references.

Relative recall is an information retrieval term and is a way of measuring recall when all relevant records are not known. When several different retrieval techniques with the same aim are combined and the relevant records are pooled, the relative recall of one specific technique is the relevant records retrieved from that technique divided by all of the pooled relevant records [15]. The relative recall technique for creating a gold standard entails using a number of published reviews, which used different retrieval techniques and work as a composite, to pool relevant records [16]. This approach is considered a more efficient way of identifying gold standard references than hand searching [16].

To limit bias, only reviews without geographically-restricted search strategies were used to create the gold standard. Reviews were selected using convenience sampling, through searching Scopus, Google Scholar and *Cochrane Database of Systematic Reviews* for systematic and scoping reviews mentioning one or more of the Nordic countries. The reviews had to be relevant to human health in a broad sense, and the selected reviews span disciplines such as epidemiology, public health, mental health, nursing and lifestyle related to health. Reviews where it was unclear if searches were limited by geographic area were excluded. The reviews were screened for references that were classified in the review as being about one or more of the Nordic countries. Pooling of studies from reviews continued until over 300 references were found, which meant that 34 reviews were included.

When using relative recall, at least 100 references are needed to validate the filter, according to Sampson et al [16]. If the validation and development sets are of equal size, the gold standard could then consist of a minimum of 200 references. Ayiku et al recommend identifying 300 references, to have some margins since all references might not be available in the database used for the filter [14].

When 34 reviews had been screened, a sufficient number of references had been found. These reviews were published between 2012–2025 and for each review, 1–49 references were relevant to the Nordic countries. The median number of references was 5 and the average 9.5. In total, 309 unique references were found, of which 288 were available in PubMed and included in the gold standard. The gold standard set consists of these 288 references, from 158 different journals and spanning the publication years 1973–2024.

Of the 288 references included in the gold standard set, 71 pertain to Denmark, 56 to Finland, 30 to Iceland, 74 to Norway and 85 to Sweden. While most of the references were relevant to only one of the countries (272 of 288 references), seven of them involved two countries, seven others involved three, one reference involved four and another one involved all five countries. Since Iceland is a smaller country than the others, with under a tenth of the population of any other country in the group, finding a similar number of references about Iceland as the other countries proved difficult. Several of the included references were relevant to more than one of the countries, and any reference specified in the reviews to concern at least one of the countries was included. References described only with supranational regions that include other countries than the Nordics were excluded, such as “Europe” or “Worldwide”.

The gold standard set was split in two equal groups using the randomizer in MS Excel (RAND), resulting in a development set and a validation set with 144 references each.

### Filter Development

Geographical filters usually include the names of countries in English, and can also include country names in other languages, names of cities and regions, public health systems and population groups in the countries. The most common search fields that are used are affiliations and subject headings, followed by titles and abstracts. Some filters also use search fields for words in names of journals, all text or keyword fields [17].

In the development of this filter, text content from titles, abstracts, journal names and affiliations were used to identify terms. The final filter includes terms searched in the affiliation field, titles and abstracts, together with Medical Subject Headings (MeSH) and terms searched in the language field. Journal names are not part of the filter, since words in journal names are not searchable in PubMed. The fields used for filter development were selected based on the most relevant fields according to Ayiku et al [14].

To identify search terms, objective methods, such as term frequency analysis, can be used. This is done in order to create an evidence-based set of terms and reduce the risk of bias [2]. Other filter developers have used subjective methods, basing the search term selection on their own knowledge, research, or expert advice [2]. The development of this filter uses a combination of the two, an approach which has been noted as a practical way of ensuring objectivity while covering more aspects than a purely objective approach [17].

**Figure 1:**
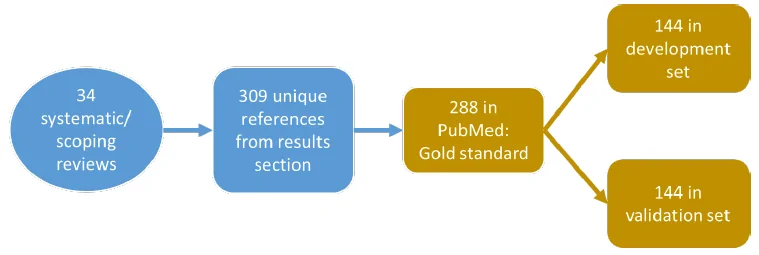
Flowchart of the gold standard, development set and validation set construction

A target corpus was created by combining the content from titles, abstracts, journal names and affiliations of all references in the development set. Using AntConc 4.2.4 [18], the target corpus was compared to the reference corpus “American English 2006: Learned” [19]. The keyword tool shows words that are statistically overrepresented (likelihood) in the target corpus compared to the reference corpus. All 485 keywords that appeared in the keyword tool were manually scanned for words related to Nordic geography and 32 terms were identified. No abbreviations were used.

The identified terms included the English names for all five countries, their adjectival forms and capital cities. All of these terms were included in the filter. Country names were also added in the countries’ main languages, as well as alternative names for the capitals where applicable. An exception was the name for Iceland in Icelandic, since “Ísland” is undistinguishable from the English word “island” when searching PubMed. Inclusion of this term would significantly worsen the filter’s precision. Names of regions comprising several of the countries were added (Scandinavia and Nordic), as well as names of autonomous regions within the countries. In addition to adjectival forms of the countries, demonyms were also added.

The remaining terms identified using the keyword tool were names of two hospitals, and 15 other names, of cities and/or municipalities and other geographic areas. Identifying all hospitals, all cities, or other such wide categories would bloat the filter and drawing the lines for what to include for each country could easily be an arbitrary decision. A category that covers almost all of these terms is “university hospitals”. 14 out of the 17 terms were covered by adding the names of university hospitals including their location. The three keywords not covered by the filter are Herlev, Gentofte and Nydalen. The first two are names of hospitals that are part of the Copenhagen University Hospital, and the last is one of the addresses of the Norwegian Institute of Public Health. None of these terms are used without other terms already included in the filter, when studied in context in the development set.

Ayiku et al recommend retrieving references by language for languages that are uncommon outside of the geographic area the filter is for [14]. This is true for the Nordic languages, and terms for the five Nordic languages that are available in PubMed were added to the filter, searched in the language field (“la”) by using the first three characters in the English name of the languages.

Two main terms in MeSH are of relevance to the Nordic countries. “Scandinavian and Nordic Countries” was introduced in 1999 and has replaced the earlier “Scandinavia”. The second main term is newer, “Scandinavians and Nordic People”, and was added to MeSH in 2023. The latter does not have any terms below it in the hierarchy, but the former includes all of the Nordic countries, with specific terms for Greenland (Denmark) and Svalbard (Norway).

The MeSH terms appearing in the development set were identified using Anne O’Tate [20]. In Anne O’Tate, the development set was imported by searching the PMIDs combined with the Boolean operator “OR”. Using the Topics tool, MeSH terms from the set were collated, and then restricted by applying the semantic filter “Geographic Areas”. The terms that were related to the Nordic countries were each country’s MeSH term. These do not have to be included explicitly, as they are all already included as terms below “Scandinavian and Nordic Countries” in the MeSH hierarchy. MeSH terms in the current filter are searched in the standard, exploded form, not excluding narrower terms below.

### Validation Methods

All records in PubMed have a unique ID. These PubMed IDs (PMIDs) were used in all validation testing as they are unambiguous, which might not be the case for titles or other information. Internal validation tests how well a filter performs on the set of references used to create it, and offers a chance to make adjustments to the filter before validating it against a new set of references that were not used during the development of the filter [17]. The development set was used to test the internal validity of the Nordic filter and recall was calculated by dividing the retrieved records with the total number of records in the development set, which solely consisted of relevant records. The missed relevant records were analyzed to see if the filter could be further improved.

External validity was assessed by testing the filter against the validation set and recall was calculated again. For both the development set and the validation set, at least 90% recall was considered sufficient, a threshold that is used in several previous studies [4, 21, 22].

**Figure 2:**
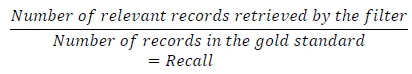
Calculation of recall

### Precision Testing Methods

Precision is the fraction of relevant retrieved references of the total retrieved references. As the gold standard is solely made up of relevant references, it cannot be used to test precision, without also identifying references that are known to be irrelevant to the filter.

A selection of the same reviews used to identify relevant references in the gold standard creation was used to test precision, since they also included references specified to be about other countries than the Nordics. One of the reviews, Balaj et al [23], was considered ineligible since its included studies were not all included in the review’s list of references. Out of the remaining reviews (n=33), five random reviews were selected using MS Excel. The references of four of these could be retrieved automatically using Scopus, while one was handled manually. 223 references were found in total, and 172 of these could be connected to a PMID with the Scopus data or manually, respectively.

The filter was tested against all PMIDs from the five reviews in PubMed. Each matching reference that was not present in the gold standard set was examined to determine if it was one of the studies that were reviewed in the results part of the review, or cited for other reasons, such as background and discussion. Since references cited for other reasons (background articles) have not been classified by country in the reviews, they were not used in the precision testing as either relevant or irrelevant.

Precision was calculated by dividing the relevant retrieved references, present in the gold standard, by all retrieved references from the results of reviews.

**Figure 3:**
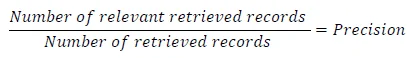
Calculation of precision

## RESULTS

### Internal Validation

Internal validation showed that the filter retrieved 136 references out of the 144 total references in the development set, or 94.4% recall. Eight references were not retrieved, two of these were missing abstracts entirely, and all but one lacked geography information in the searchable fields. The reference that could have been retrieved by a filter included a city and its county in the affiliation field of an author (Gävle and Gävleborg). To balance recall and precision, cities and counties have not been added to this filter. No changes were made to the filter as a result of internal validity testing. External validation followed as the threshold recall of 90% was surpassed during internal validation.

### External Validation

External validation using the validation set showed that 139 references out of the total 144 were retrieved by the filter. This means that the recall was 96.5%. The five missed references in the validation set all lacked affiliation information and one of them did not have an abstract. None of them used any geography terms in their abstracts and only one had a MeSH term related to geography. As it was “Europe”, it was too broad to qualify for a Nordic countries filter.

**Table 1:** Final filter

Line	Search string	Notes
1	“Scandinavians and Nordic People”[mh] OR “Scandinavian and Nordic Countries”[mh]	MeSH related to the Nordic countries
2	scandinavia*[tiab] OR nordic*[tiab] OR denmark[tiab] OR finland[tiab] OR iceland[tiab] OR norway[tiab] OR sweden[tiab] OR danmark[tiab] OR suomi[tiab] OR norge[tiab] OR noreg[tiab] OR sverige[tiab] OR aland[tiab] OR faroe*[tiab] OR greenland*[tiab] OR scandinavia*[ad] OR nordic*[ad] OR denmark[ad] OR finland[ad] OR iceland[ad] OR norway[ad] OR sweden[ad] OR danmark[ad] OR suomi[ad] OR norge[ad] OR noreg[ad] OR sverige[ad] OR aland[ad] OR faroe*[ad] OR greenland*[ad]	Terms for countries, supranational regions and autonomous regions, searched in titles, abstracts and affiliations
3	danish[tiab] OR finnish[tiab] OR icelandic[tiab] OR norwegian[tiab] OR swedish[tiab] OR danes[tiab] OR finns[tiab] OR icelanders[tiab] OR norwegians[tiab] OR swedes[tiab] OR alanders[tiab] OR danish[ad] OR finnish[ad] OR icelandic[ad] OR norwegian[ad] OR swedish[ad] OR danes[ad] OR finns[ad] OR icelanders[ad] OR norwegians[ad] OR swedes[ad] OR alanders[ad]	Adjectival forms and demonyms, searched in titles, abstracts and affiliations
4	copenhagen[tiab] OR helsinki[tiab] OR helsingfors[tiab] OR kobenhavn[tiab] OR reykjavik[tiab] OR oslo[tiab] OR stockholm[tiab] OR copenhagen[ad] OR helsinki[ad] OR helsingfors[ad] OR kobenhavn[ad] OR reykjavik[ad] OR oslo[ad] OR stockholm[ad]	Capital cities searched in titles, abstracts and affiliations
5	aalborg[tiab] OR aarhus[tiab] OR abo[tiab] OR akademiska[tiab] OR akershus[tiab] OR bergen[tiab] OR goteborg[tiab] OR gothenburg[tiab] OR haukeland[tiab] OR huddinge[tiab] OR karolinska[tiab] OR kuopio[tiab] OR landspitali[tiab] OR linkoping[tiab] OR lorenskog[tiab] OR lund[tiab] OR malmo[tiab] OR norrland*[tiab] OR odense[tiab] OR orebro[tiab] OR oulu[tiab] OR rigshospitalet[tiab] OR roskilde[tiab] OR sahlgrenska[tiab] OR sjaelland*[tiab] OR skane*[tiab] OR solna[tiab] OR “st olav*”[tiab] OR stavanger[tiab] OR tammerfors[tiab] OR tampere[tiab] OR tromso[tiab] OR trondheim[tiab] OR turku[tiab] OR uleaborg*[tiab] OR umea[tiab] OR uppsala[tiab] OR aalborg[ad] OR aarhus[ad] OR abo[ad] OR akademiska[ad] OR akershus[ad] OR bergen[ad] OR goteborg[ad] OR gothenburg[ad] OR haukeland[ad] OR huddinge[ad] OR karolinska[ad] OR kuopio[ad] OR landspitali[ad] OR linkoping[ad] OR lorenskog[ad] OR lund[ad] OR malmo[ad] OR norrland*[ad] OR odense[ad] OR orebro[ad] OR oulu[ad] OR rigshospitalet[ad] OR roskilde[ad] OR sahlgrenska[ad] OR sjaelland*[ad] OR skane*[ad] OR solna[ad] OR “st olav*”[ad] OR stavanger[ad] OR tammerfors[ad] OR tampere[ad] OR tromso[ad] OR trondheim[ad] OR turku[ad] OR uleaborg*[ad] OR umea[ad] OR uppsala[ad]	Names of university hospitals and their locations, searched in titles, abstracts and affiliations
6	dan[la] OR fin[la] OR ice[la] OR nor[la] OR swe[la]	Nordic languages, searched in the language field
7	#1 OR #2 OR #3 OR #4 OR #5 OR #6	Combination of all lines

**Table 2:** Internal validation

References	Number	Percentage of development set
Development set	144	100%
References retrieved	136	94.4%
References not retrieved	8	5.6%
Not retrieved, without geographic information	7	4.9%
Not retrieved, with geographic information	1	0.7%

**Table 3:** External validation

References	Number	Percentage of validation set
Validation set	144	100%
References retrieved	139	96.5%
References not retrieved	5	3.5%
Not retrieved, without geographic information	4	2.8%
Not retrieved, with geographic information	1	0.7%

### Precision

The references of five reviews were examined to determine the filter’s precision. 172 references with PMIDs were identified and 33 of these were retrieved by the filter. After removing references that were part of the gold standard set, 10 references remained that were retrieved by the filter. All but one were cited as background or for discussion purposes. A single reference was a false positive. It was part of the results of a review and classified there as not being about the Nordic countries, but was still retrieved by the filter. The reason was that one of the authors was affiliated with a Swedish university. Since one false positive was found out of the 24 references that matched the filter and were not background articles, the precision was 95.8%, or 23 relevant references out of 24 retrieved.

## DISCUSSION

A balanced filter was created, with high recall and precision. Recall was tested on 288 references in total, and only one reference that could have been retrieved by a filter was missed.

This filter was developed for PubMed and is meant for use in searches related to human health. Geographic filters are often developed for use in health care resources, although they may not be designed for PubMed [17]. Systematic searching is common in evidence-based medicine, but also used in many other fields. In disciplines like the social sciences and in education research, a filter for the Nordic countries could also be of use, since there are similar societal institutions in the region. A valuable direction for future research would be the development of filters for this region in other health care databases as well as for databases for other disciplines.

Even though the recall was proven acceptable in this study, the filter should not be considered “highly sensitive”, since the aim was to develop a balanced filter. Ideally, a recall-maximizing filter should be developed and tested on an even larger number of references, where the 100 reference threshold could be surpassed per country. Gathering a large volume of references is a demanding task, and no Nordic organization is currently responsible for developing and validating search filters. Several existing geographic filters for other regions are developed by the United Kingdom’s National Institute for Health and Care Excellence (NICE) where NICE guidelines were used to develop gold standard sets [3, 5, 24]. There is no corresponding institution for the Nordic countries, but one or more national institutions, for example the fairly similar institutes for public health [25], could be in an advantageous position when it comes to this type of development.

Other geographic filters have used the Boolean operator “NOT”, which is not used in this filter. In the UK filters for Medline and Embase, locations containing UK related terms were excluded, such as “New England” and “British Columbia” [3, 26]. Similar uses are in the filters for Spain and Africa [27, 28]. Similar issues arise with the Nordic terms, for example the Nordic diet, Nordic walking, and the Nordic hamstring exercise, and several combinations with other more local geographic terms such as the Declaration of Helsinki or the ABO blood group system (which has nothing to do with Åbo/Turku, Finland). For the present filter, these issues were not deemed serious enough to warrant phrase exclusion, since the terms are not part of geographic names from the wrong part of the world and do not seem to be common phrases across many fields of health research.

Users of the filter can of course append exclusions of specific phrases if necessary for the specific topic that the filter is used in combination with. A systematic literature search on sleep in the Nordic countries could for example choose to exclude “Karolinska sleepiness scale” from “Karolinska” in titles and abstracts, while making sure to flag any such changes as their own alteration to the validated filter.

Some issues are impossible to avoid, such as geographical terms as last names, most notably “Lund”. This was also noted by the developers of the UK filters, as well as the reduced precision caused by using the affiliation field, since authors don’t necessarily write about the same country where they are affiliated [3, 26].

It is worth mentioning that the filter currently incites an error message in PubMed since some of the terms are not indexed in any references yet. This does not affect the search and the error message requires no action. If PubMed references are added in the future where these terms are present, they will be captured by the filter.

This filter is the first publicly available, validated filter for the Nordic countries. It can be used by information specialists, health librarians, health researchers and professionals in various health related fields who aim to collect evidence from the Nordic countries related to human health. This freely available filter for a database that is also free to search means that health care practitioners in diverse circumstances can find evidence with relevance to the Nordic countries efficiently. For many librarians and information specialists, this validated filter will likely increase the recall of search strategies compared to ad hoc solutions. Ultimately, the consequences are that fewer relevant references are missed and that systematic searches related to the Nordic countries give a more complete picture of the available research. Using the filter will reduce the risk of missing relevant studies and save resources since it can be reused in many different contexts.

## Data Availability

Data associated with this article are available in the Open Science Framework at https://osf.io/6ktva.

## Footnotes

* The Core Collection used covers the Science Citation Index, Social Sciences Citation Index, Arts & Humanities Citation Index, Emerging Sources Citation Index, Conference Proceedings Citation Index and Book Citation Index.

† In PubMed: (nordic*[tiab] OR scandinavia*[tiab]) AND (filter*[ti] OR hedge*[ti] OR “search strateg*”[ti]); in Scopus: TITLE-ABS-KEY(nordic* OR scandinavia*) AND TITLE(filter* OR hedge* OR “search strateg*”); and in Web of Science: TS=(nordic* OR scandinavia*) AND TI=(filter* OR hedge* OR “search strateg*”)

## SUPPLEMENTAL FILES

**Appendix A:** Executable Search Filter

**Appendix B:** Reviews

**Appendix C:** Unique Papers Identified from Reviews

**Appendix D:** Antconc Keywords

**Appendix E:** Gold Standard Papers

**Appendix F:** Read Me
